# Interventions Related to Menstrual Health and Menstrual-Cycle-Associated Symptoms in Female Athletes: Implications for Recovery and Athletic Performance

**DOI:** 10.3390/sports14060236

**Published:** 2026-06-08

**Authors:** Nina Mendez-Dominguez, Damaris Estrella-Castillo, Edgar Villarreal-Jimenez, Russell Arcila-Novelo

**Affiliations:** 1 Hospital Regional de Alta Especialidad de la Peninsula de Yucatan, Servicios de Salud del Instituto Mexicano del Seguro Social para el Bienestar IMSS-BIENESTAR, Merida 97130, Mexico; a23220303@alumnos.uady.mx; 2 Facultad de Medicina, Universidad Autonoma de Yucatan, Merida 97000, Mexico; ecastill@correo.uady.mx (D.E.-C.); arcinove@correo.uady.mx (R.A.-N.)

**Keywords:** menstrual cycle, athletes, drug therapy, physical fitness, systematic review

## Abstract

Background: Menstrual-cycle-associated symptoms and menstrual health conditions are common among female athletes and may influence recovery, perceived readiness, training availability, and athletic performance. However, evidence regarding interventions aimed at managing these symptoms and their functional implications in athletes remains limited and heterogeneous. Objective: The objective of this study is to synthesize the available evidence on pharmacological and non-pharmacological interventions related to menstrual health and menstrual-cycle-associated symptoms in female athletes and to evaluate their impact on performance, recovery, functional capacity, and symptom burden. Materials and Methods: A systematic review with narrative synthesis was conducted following PRISMA 2020 guidelines. Studies evaluating interventions associated with menstrual health or menstrual-cycle-related symptoms in female athletes were included when they reported outcomes related to athletic performance, recovery, functional capacity, or symptom burden. Results: Five studies published between 2023 and 2025 were included. The interventions evaluated included hormonally related strategies involving oral contraceptive timing, recovery interventions such as cryotherapy, mindfulness-based yoga, nutritional supplementation, and pharmacological pain-modulation approaches. However, findings regarding objective athletic performance outcomes were inconsistent, and the included studies showed substantial methodological heterogeneity. Conclusions: The available evidence suggests that certain interventions related to menstrual health may contribute to improvements in symptom burden, perceived recovery, or selected functional outcomes in female athletes. Nevertheless, the current evidence base remains limited, heterogeneous, and insufficient to support strong performance-related recommendations. Further high-quality studies specifically designed in female athlete populations are needed to inform evidence-based sports medicine practice.

## 1. Introduction

In the last two and a half decades, in parallel with the beginning of the 21st century, women’s participation in sport has experienced a sustained increase on a global scale. This growth has been accompanied by an intensification of the physical, psychological and social demands placed on female athletes. In sport scenarios characterized by high training loads, congested competition calendars, extensive travel demands, and often limited recovery opportunities, performance preservation depends on rigorous management of recovery, stress, and overall health [1]. Despite this quantitative and qualitative advancement of women’s sport, scientific evidence underpinning contemporary training, recovery and performance optimization strategies continues to originate disproportionately in male or mixed cohorts. This methodological asymmetry raises substantive questions regarding the external validity and applicability of such approaches in female athlete populations [2,3].

It has been consistently documented that women continue to be underrepresented as participants in exercise science and sports medicine studies, a situation largely attributable to the historical prioritization of research focused on male athletes or mixed samples. This bias persists even though systematic consideration of the cyclical physiological characteristics of female athletes could guide the development of specific strategies aimed at improving their health and well-being, with potential concomitant benefits on health, well-being, and athletic performance. Women frequently represent less than one third of study participants in many sports science journals in the last decade [2,4] which translates into unmet knowledge needs of relevance to sports medicine. This evidence gap limits the formulation of specific recommendations for women and favors the extrapolation of findings derived from male populations, despite the existence of well-established biological differences that can modulate training adaptation, recovery processes, and performance outcomes [3].

Among the physiological characteristics that modulate body experience and performance in women, both athletes and non-athletes, the menstrual cycle is a central and distinctive factor. In eumenorrheic women who do not use hormonal contraceptives, estrogen and progesterone concentrations fluctuate predictably throughout the menstrual cycle and exert regulatory effects on multiple physiological systems beyond reproductive function. These include, but are not limited to, energy metabolism, neuromuscular function, thermoregulation, perception of exertion, and recovery processes, with potential implications for performance and competitiveness [5,6]. From a biological perspective, estrogen has been proposed to influence endurance-related physiological processes through modulation of carbohydrate, fat, and protein metabolism, although the practical effects on athletic performance remain inconsistent across studies [5,6].

The available evidence indicates that variations in physical performance throughout the menstrual cycle are heterogeneous. While some studies have reported differences in endurance or potency tests between cycle phases, others have not identified significant variations in target performance [5,7]. This inconsistency has been attributed, in part, to methodological differences, variability in hormonal fluctuations, and the use of indirect methods to determine menstrual phase [6]. On the other hand, the subjective perception of performance and effort shows more consistent patterns, with a greater report of reduced perceived performance during the early follicular phase and the late luteal phase [6,7].

Beyond hormonal fluctuations themselves, female athletes frequently experience menstrual-cycle-associated symptoms that may affect training quality, recovery, competitive readiness, and perceived performance. Commonly reported symptoms include dysmenorrhea, fatigue, bloating, sleep disturbances, headaches, gastrointestinal symptoms, mood changes, and impaired concentration, particularly during the late luteal and early follicular phases of the cycle [7,8,9,10]. Observational studies in exercising women have shown that these symptoms are associated with reduced availability to train and compete, altered recovery behaviors, and decreased perceived athletic performance [9]. In response, athletes frequently employ pharmacological and non-pharmacological self-management strategies including analgesics, hormonal contraceptives, nutritional approaches, recovery interventions, mindfulness practices, and training modifications, although the evidence supporting these interventions remains limited [10].

Among menstrual-cycle-associated symptoms, heavy menstrual bleeding deserves particular attention in sports medicine because of its potential relationship with fatigue, iron deficiency, impaired recovery, and reduced training tolerance. Heavy menstrual bleeding (menorrhagia) is currently defined as excessive menstrual loss that adversely affects women’s physical, social, emotional, and/or material quality of life, regardless of objectively measured menstrual volume [11]. This approach focused on functional impact has progressively replaced strictly volumetric definitions, recognizing that subjective burden and clinical consequences are key determinants for therapeutic decision-making [11] and to avoid dismissing the phenomenon that presents interpersonal variability based on a reference measurement. In general populations, heavy menstrual bleeding is estimated to affect approximately one in five women of reproductive age and is associated with iron-deficiency anemia, persistent fatigue, pain, and impaired psychosocial well-being [11,12].

Observational studies and surveys in athletic populations show that approximately 30% to 40% of elite female athletes report heavy menstrual bleeding, with similar or even higher prevalences in endurance sports [9,10]. This condition has been associated with iron deficiency, lower training tolerance, increased perception of fatigue, and reduced availability to train or compete. Despite its frequency and potential impact on health and performance, menstrual bleeding and its management strategies are rarely systematically evaluated in research in, let alone evidence-based recommendations for athletes [10].

Menstrual dysfunctions are closely related to energy availability and training load, particularly in disciplines sensitive to body weight, of an aesthetic nature or with high volumes of practice. Recent evidence in female athletes indicate that amenorrhea and other clinically relevant alterations of the menstrual cycle are common in association to low energy availability, higher training volumes, and a higher incidence of injury-related sports absences [13]. These findings reinforce that menstrual disturbances are not only a marker of health risk, but also a factor that can compromise training continuity and long-term performance sustainability.

From a clinical, therapeutic, and allopathic perspective, there are multiple nonsurgical options for the management of heavy menstrual bleeding and other menstrual symptoms in women of reproductive age, including nonsteroidal anti-inflammatory drugs, hormonal contraceptives, progestogens, and antifibrinolytic agents. These interventions have been shown to be effective in reducing bleeding and improving quality of life in general gynecologic and adolescent populations [11,12], these interventions also require consideration of training adaptation, competition scheduling, recovery demands, tolerability, and compliance with anti-doping regulations.

In the context of sports, the application of pharmacological interventions poses additional challenges related to safety, potential interference with physiological adaptation, inter-individual variability of responses, and anti-doping regulatory considerations. In sports, both pharmacological and non-pharmacological strategies are employed for the management of menstrual symptoms, including modification of training load, physical recovery interventions, mind–body practices, nutritional optimization, and menstrual health education [1,8]. However, the effects of these interventions on specific performance outcomes in female athletes deserve to be synthesized systematically. Existing reviews have predominantly focused on the effects of the menstrual cycle on performance or in non-athletic populations and rarely evaluate interventions explicitly aimed at symptom management with athletic performance-related outcomes [5,6]. Tracking symptoms associated with menstrual cycle can help athletes be aware of patterns and incorporate methods for mitigating or alleviating the symptoms [14]. This knowledge gap underscores the need to critically integrate the available evidence, identify relevant research gaps, and guide future scientific agendas focused on women’s health and performance in sports.

Within this context, menstrual symptom monitoring may also represent an important component of athlete health surveillance, readiness assessment, fatigue management, and individualized recovery strategies in female athletes.

In this context, the aim of this systematic review is to synthesize the available evidence on pharmacological and non-pharmacological interventions related to menstrual health and menstrual-cycle-associated symptoms in female athletes, and to evaluate their potential effects on athletic performance, recovery, functional capacity, and symptom burden. Additionally, this review seeks to identify current evidence gaps and inform future research and sports medicine practice focused on women’s health in sports.

## 2. Materials and Methods

The review protocol was registered in PROSPERO CRD420251275744. The present study corresponds to a systematic review of the literature with narrative synthesis, developed following the methodological principles established in the PRISMA 2020 guide for systematic reviews [15].

Eligibility criteria. Inclusion and exclusion criteria were defined a priori using the PICO framework (Population, Intervention, Comparator, Outcomes). Athletes were operationally defined as women participating in structured training and organized competition at collegiate, regional, national, or international levels. Eligible studies were those including eumenorrheic women and/or users of hormonal contraceptives; hormonal contraceptive use was extracted and reported descriptively because of its potential influence on menstrual-related outcomes. We excluded studies conducted exclusively in recreationally active or non-athlete populations without organized competitive participation.

Interventions. Pharmacological interventions included analgesics, hormonal interventions, or nutritional supplementation when used with therapeutic intent for menstrual-related symptoms. Non-pharmacological interventions included training modifications, recovery interventions, mindfulness-based approaches, and other behavioral or physical strategies. We accepted studies with Control group, Intra-individual comparison, Comparison between phases of the menstrual cycle or Comparison with usual training or management.

Outcomes evaluated. The primary outcomes included objective or sport-specific measures of athletic performance and functional capacity, such as strength, endurance, aerobic performance, reactive force indices, or sport-specific functional tests. With respect to study types, we included both randomized controlled trials and crossover studies. We excluded narrative or systematic reviews, protocols with no published results, observational studies without intervention, and grey literature (thesis, conference proceedings, technical reports).

Systematic search was conducted in PubMed/MEDLINE, EMBASE, and Web of Science from database inception through 28 December 2025. The final search update was performed on December 2025. The search strategy combined controlled terms (MeSH and Emtree) and free text related to female athletes’ menstrual cycle; menstrual or premenstrual symptoms; pharmacological and non-pharmacological interventions and finally, sports performance. The string search strategy included combinations of the terms (‘female athletes’ OR ‘women athletes’) AND (‘menstrual cycle’ OR ‘menstrual symptoms’ OR ‘premenstrual symptoms’) AND (‘performance’ OR ‘recovery’ OR ‘exercise performance’) in English or Spanish. All retrieved records were imported into a reference manager for automatic review and duplicate verification. Subsequently, two screening stages were performed:

1. Review of titles and abstracts, to exclude clearly irrelevant studies.
2. Full-text review, to confirm final eligibility.

Two reviewers independently screened titles and abstracts, followed by full-text assessment for eligibility. Discrepancies were resolved through consensus discussion. A standardized database was designed to systematically record extracted data from the included studies. The extracted data included study design, participant characteristics, menstrual status, intervention type, comparator, outcomes assessed, main findings, and risk-of-bias information.

These databases were selected because together they provide broad coverage of biomedical, clinical, exercise science, and sports medicine literature, while minimizing duplicate retrieval of records across highly overlapping sources. Given the focused scope of interventions related to menstrual health and athletic performance, PubMed/MEDLINE, EMBASE, and Web of Science were considered sufficient to capture the relevant peer-reviewed literature. Nevertheless, omission of additional databases such as SPORTDiscus, CINAHL, or the Cochrane Central Register of Controlled Trials may have resulted in missed studies and should be considered a limitation of this review.

**Figure 1:**
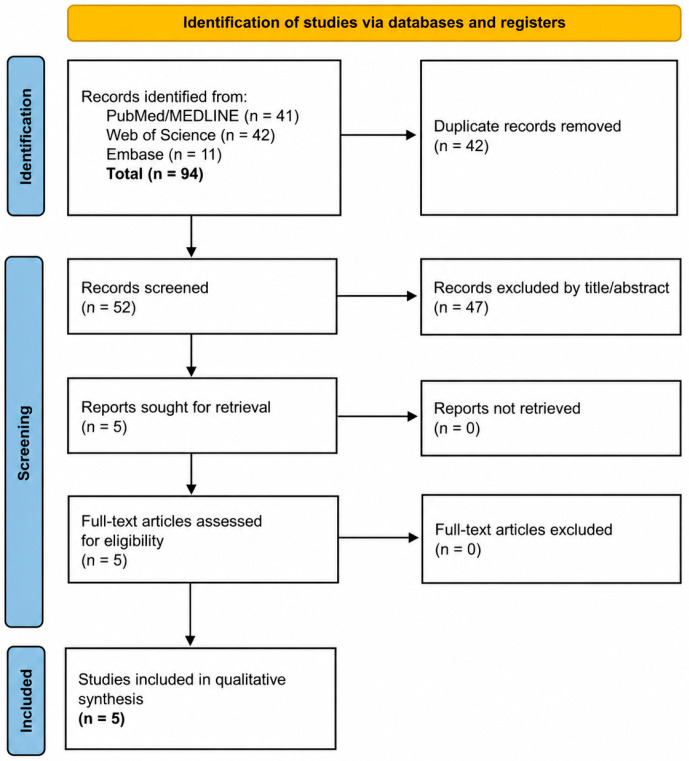
PRISMA 2020 study selection flow diagram.

Both the methodological quality and risk of bias of the included studies were assessed using validated tools according to the study design, prioritizing domains of (1) Generation and concealment of assignment, (2) Blinding, (3) Management of incomplete data, (4) Selectivity in the reporting of results, and (5) global risk using the RoB2 tool [16]. Given the substantial methodological heterogeneity, small number of studies, and narrative nature of the synthesis, a formal certainty-of-evidence assessment using GRADE was not performed [17]. Due to heterogeneity in interventions, study designs, and outcome measures, a quantitative meta-analysis was not feasible. Therefore, findings were synthesized narratively according to intervention type, symptom-related outcomes, recovery-related outcomes, and performance-related outcomes. The selection process is presented in the PRISMA flowchart in Figure 1 and Prisma checklist as Supplementary Table S1.

## 3. Results

Five studies published between 2024 and 2025 met the inclusion criteria and were included in the final synthesis. The studies were conducted in Europe, North America, and the Middle East and included female athletes participating in endurance training, strength training, combat sports, football, and CrossFit^®^ disciplines. The interventions evaluated included hormonally related interventions involving oral contraceptive timing, cryotherapy, mindfulness-based yoga, nutritional supplementation, and pharmacological interventions targeting pain modulation and recovery. Considerable heterogeneity was observed in study designs, participant characteristics, interventions, and outcome measures, precluding quantitative meta-analysis. Although some included studies primarily evaluated recovery, hormonal context, or performance-related outcomes rather than direct symptom treatment, they were included because they assessed interventions potentially related to menstrual health, menstrual-cycle-associated symptom burden, perceived recovery, or functional readiness in female athletes.

Summary of findings. The database search identified 94 records, including 41 from PubMed/MEDLINE, 42 from Web of Science, and 11 from EMBASE. After removal of 42 duplicate records, 52 studies remained for title and abstract screening. Following screening, five full-text articles were assessed for eligibility, and all met the inclusion criteria for qualitative synthesis. The study selection process is summarized in the PRISMA flow diagram (Figure 1).

The characteristics of the included studies are summarized in Table 1. The studies included female participants engaged in structured physical training or athletic activity, with sample sizes ranging from 15 to 29 participants. Three studies included naturally menstruating or eumenorrheic athletes, while one specifically evaluated users of monophasic oral contraceptives and another included athletes evaluated during different menstrual phases. All five studies used randomized controlled or randomized crossover designs.

Regarding intervention type, two studies evaluated pharmacological or hormonally related interventions, while three assessed non-pharmacological approaches. The pharmacological or hormonal studies evaluated acute acetaminophen ingestion and oral contraceptive timing, respectively. Non-pharmacological interventions included total-body cryotherapy, mindfulness-based yoga, and dark chocolate supplementation.

The study by Bisgaard et al. [18] analyzed whether the timing of oral contraceptive intake influenced physical performance-related parameters in recreationally active women using monophasic oral contraceptives. Small but significant differences were observed in balance time and handgrip strength depending on oral contraceptive timing, while no substantial effects were identified in aerobic capacity, countermovement jump performance, agility, flexibility, or maximal oxygen consumption.

Three studies evaluated non-pharmacological interventions related to menstrual health, recovery, or symptom burden. Messaoudène et al. [19] investigated the effects of total-body cryotherapy and reported improvements in fatigue-related and recovery-related parameters, including perceived fatigue and sleep-related outcomes, although no consistent improvements in objective athletic performance were observed.

SantaBarbara et al. [20] conducted a randomized crossover trial evaluating a brief daily mindfulness yoga intervention in female athletes. The intervention was associated with improvements in perceived performance, stress reduction, and menstrual-related symptoms, particularly during phases associated with greater symptom burden, although objective physical performance metrics remained unchanged.

Safari et al. [21] evaluated acute dark chocolate supplementation in female CrossFit^®^ athletes and observed improvements in functional performance during the CINDY WOD test, together with favorable effects on cognition and delayed-onset muscle soreness. These effects appeared more evident during menstrual phases associated with increased symptom perception.

BenSalem et al. [22] evaluated the acute effects of acetaminophen ingestion on repeated high-intensity exercise performance and recovery-related outcomes in well-trained female athletes. Acetaminophen administration was associated with improvements in repeated sprint performance, attention, mood state, perceived recovery, and pain-related outcomes, while reductions in perceived exertion and delayed-onset muscle soreness were also reported.

Clinical implications. Across studies, symptom burden, pain perception, fatigue, delayed-onset muscle soreness, stress, perceived recovery, and perceived performance were frequently assessed as secondary outcomes. Improvements were more consistently observed in subjective or recovery-related outcomes than in objective athletic performance measures. In particular, reductions in perceived pain, stress, fatigue, and muscle soreness were reported in several studies, especially during symptomatic phases of the menstrual cycle.

Assessment of methodological quality and risk of bias (Table 2) showed that two studies were classified as having a low risk of bias [18,21], while three presented moderate a risk of bias [19,20,22]. The principal limitations identified included small sample sizes, heterogeneity in outcome assessment, and challenges related to blinding procedures inherent to several interventions. No studies were excluded on the basis of methodological quality in order to preserve transparency in study selection and synthesis of the available evidence.

Findings suggest that both pharmacological and non-pharmacological interventions related to menstrual health, symptom burden, recovery, or performance optimization may contribute to improvements in selected functional or perceptual outcomes in female athletes. However, the available evidence remains limited, methodologically heterogeneous, and insufficient to support definitive recommendations regarding objective athletic performance outcomes.

## 4. Discussion

This systematic review synthesized five studies on pharmacological and non-pharmacological interventions related to menstrual health, menstrual-cycle-associated symptoms, recovery, and performance-related outcomes in female athletes. Although the number of included studies was limited, the findings allowed identification of emerging patterns, important evidence gaps, and priority areas for future research in sports medicine focused on women’s health and athletic performance.

Summary of findings. A central finding of this review is that the effects observed on athletic performance were heterogeneous and strongly dependent on the intervention type, outcome evaluated, menstrual or hormonal context, and methodological design of each study. This pattern is consistent with previous reviews reporting inconsistent associations between menstrual-cycle phases and objective performance outcomes, despite the existence of well-described physiological mechanisms involving hormonal fluctuations [5,7]. The available evidence suggests that menstrual-cycle-associated interventions may exert greater influence on symptom burden, perceived recovery, and subjective readiness to train or compete than on objective performance metrics such as aerobic capacity, maximal strength, or power output.

The distinction between objective and perceived performance emerged as a particularly relevant finding. Several studies did not demonstrate statistically significant improvements in objective indicators of performance, while simultaneously reporting reductions in stress, pain, fatigue, muscle soreness, or perceived exertion [19,20,21]. This observation is consistent with previous literature showing that female athletes frequently report reduced perceived performance during the early follicular and late luteal phases, even when objective performance changes are inconsistent or clinically modest [1,5,8]. In elite sport settings, where training availability, recovery quality, and readiness to compete are highly relevant, these subjective improvements may nevertheless have important practical implications.

Non-pharmacological interventions. Among the non-pharmacological interventions evaluated, mindfulness-based yoga, cryotherapy, and nutritional supplementation demonstrated potentially favorable effects on recovery-related and symptom-related outcomes. SantaBarbara et al. [20] reported improvements in perceived performance, stress reduction, and menstrual-related symptoms following a brief mindfulness yoga intervention. Similarly, Messaoudène et al. [19] observed improvements in fatigue-related and recovery-related parameters after total-body cryotherapy, although no consistent effects were identified in objective athletic performance. Safari et al. [21] found that acute dark chocolate supplementation was associated with improvements in functional performance, cognition, and delayed-onset muscle soreness in female CrossFit^®^ athletes, particularly during menstrual phases associated with increased symptom perception.

These findings suggest that female athletic performance does not respond in a simple linear manner to isolated hormonal variations or single interventions, but rather reflects complex interactions among physiological status, symptom expression, recovery, psychological factors, and training demands [1,5]. They also support the importance of integrating symptom burden and recovery outcomes into sports medicine research involving female athletes, rather than focusing exclusively on traditional performance metrics.

Pharmacological interventions. The pharmacological and hormonally related studies included in this review also provide relevant insights, although their findings should be interpreted cautiously. Bisgaard et al. [18] evaluated whether the timing of oral contraceptive intake influenced physical performance-related parameters in recreationally active women using monophasic oral contraceptives. Small differences were observed in balance and handgrip strength depending on oral contraceptive timing, while most objective performance measures remained unchanged. These findings suggest that acute exogenous hormonal exposure may influence selected physiological or neuromuscular parameters, although the overall effects on athletic performance appear limited.

BenSalem et al. [22] demonstrated that acute acetaminophen ingestion improved repeated high-intensity sprint performance, mood state, attention, perceived recovery, and pain-related outcomes in well-trained female athletes. However, this intervention was not specifically designed to treat menstrual symptoms, and its results should therefore be interpreted primarily in the context of pain modulation, exercise tolerance, and recovery-related outcomes. The study nevertheless highlights how pain-related interventions may influence both perceptual and functional responses in female athletes during periods of increased symptom burden.

Clinical implications. From a broader clinical perspective, management of heavy menstrual bleeding and menstrual symptoms remains highly relevant in female athletes because of its potential relationship with iron deficiency, fatigue, impaired recovery, and reduced training tolerance [11,12]. Contemporary gynecologic literature supports the use of nonsteroidal anti-inflammatory drugs, hormonal contraceptives, progestogens, and antifibrinolytic agents as effective first-line approaches for symptom control and quality-of-life improvement in general and adolescent populations [11,12]. However, the present review demonstrates that direct evidence evaluating these interventions specifically in athletes remains scarce.

This gap between gynecological evidence and sports medicine evidence has important implications. Female athletes may experience unique physiological demands, training loads, recovery requirements, and competitive pressures that could modify both symptom perception and intervention responses [23,24]. Additionally, normalization of menstrual symptoms within sport environments may contribute to underdiagnosis, undertreatment, and insufficient integration of menstrual health into athletic care [24,25]. Consequently, extrapolation from general gynecological populations to female athletes should be approached cautiously until more athlete-specific evidence becomes available.

Limitations and future directions. The methodological limitations of the current evidence base deserve emphasis. Only five studies met eligibility criteria, increasing the possibility of publication bias and limiting the robustness of the available evidence. Furthermore, all included studies were published between 2024 and 2025, reflecting the emerging nature of this field but also restricting the temporal breadth of the evidence base. The included studies were characterized by small sample sizes, heterogeneous methodologies, variable definitions of menstrual phases, inconsistent hormonal verification methods, and diverse performance outcomes, all of which limited comparability across studies and precluded quantitative meta-analysis. In addition, athlete populations were limited in size and diversity, with findings derived primarily from specific sports and training contexts, reducing generalizability to the broader population of female athletes. Finally, caution is warranted when extrapolating findings from specific interventions to broader categories of menstrual health management because the interventions evaluated differed substantially in mechanism, duration, and intended outcomes.

Despite these limitations, the present review contributes to an emerging field of sports medicine research focused on women’s health and highlights the need for more rigorous and athlete-centered investigation. Future studies should incorporate standardized assessment of menstrual symptoms, objective hormonal verification, sport-specific performance outcomes, and functional measures related to recovery and training availability. Additionally, larger randomized trials evaluating both pharmacological and non-pharmacological interventions in diverse athletic populations are needed to establish evidence-based recommendations for menstrual health management in sport.

## 5. Conclusions

The limited evidence currently available does not indicate an absence of effect, but rather an absence of directed research evaluating interventions related to menstrual health and menstrual-cycle-associated symptoms in female athletes. This finding highlights a substantial gap in the sports medicine literature and underscores the need for greater attention to menstrual health as a component of athlete care and performance optimization.

The evidence synthesized in this review suggests that certain interventions related to menstrual health, symptom burden, recovery, or performance optimization may contribute to improvements in perceived recovery, pain, fatigue, stress, and selected functional outcomes in female athletes. Non-pharmacological interventions such as mindfulness-based practices, cryotherapy, and nutritional supplementation demonstrated potentially favorable effects on well-being, recovery-related outcomes, and perceived performance in specific contexts. However, findings regarding objective measures of athletic performance remain inconsistent and insufficient to support definitive performance-related recommendations.

These findings should be interpreted considering the important methodological limitations of the available evidence, including the small number of studies, limited athlete populations, heterogeneous study designs, variability in menstrual phase assessment and hormonal verification, and the diversity of interventions and performance outcomes evaluated. Consequently, the current evidence base does not permit firm conclusions regarding the comparative effectiveness of specific pharmacological or non-pharmacological strategies.

Overall, the available literature supports the importance of integrating menstrual health and menstrual-cycle-associated symptoms into sports medicine research, athlete monitoring, and individualized recovery management. Routine assessment of menstrual symptoms, perceived recovery, fatigue, training tolerance, and readiness to compete may help identify opportunities to optimize athlete well-being and performance. Future research should incorporate standardized hormonal assessment methods, sport-specific performance outcomes, and larger, more diverse athlete populations to generate robust evidence-based recommendations for menstrual health management in sport.

## Figures and Tables

**Table 1:** Characteristics of the studies included (*n* = 5) in the systematic review.

Author (Year)	Country	Study Design	Population/Sport	Sample Size	Menstrual/Hormonal Context	Intervention	Comparator	Primary Performance Outcomes	Secondary Outcomes	Main Findings
Bisgaard [18]	Denmark	Randomized crossover-controlled study	Recreationally active women using monophasic oral contraceptives	*n* = 29	Oral contraceptive users	Oral contraceptive intake 1 h before testing	Oral contraceptive intake 24 h before testing	Balance, handgrip strength, VO_2_ peak, agility, countermovement jump	Psychological well-being, pain, motivation, lactate, flexibility	Minor differences were observed in balance and handgrip strength depending on oral contraceptive timing, while most performance variables were unchanged.
Messaoudène [19]	France	Randomized controlled crossover trial	Young female athletes	*n* = 17	Menstrual cycle monitored	Total body cryotherapy	Hands-off cycle control	Fatigue and functional performance	Sleep quality, heart rate variability, body temperature	Cryotherapy was associated with improvements in fatigue-related and recovery-related parameters.
Santa Barbara [20]	United States	Randomized crossover trial	Women endurance/strength athletes	*n* = 20 (18 final analysis)	Naturally menstruating athletes	Daily mindfulness yoga (10 min/day)	Cycle without intervention	Perceived performance	Menstrual symptoms, stress, rating of perceived exertion	Mindfulness yoga was associated with improvements in perceived performance, stress, and menstrual-related symptoms.
Safari [21]	Iran	Randomized double-blind crossover trial	Female CrossFit^®^ athletes	*n* = 15	Menstrual cycle monitored	Dark chocolate 85% (30 g/day for 3 days)	Placebo and control	Functional performance (CINDY WOD)	Cognition (Stroop test), delayed-onset muscle soreness	Dark chocolate supplementation was associated with improvements in functional performance, cognition, and delayed-onset muscle soreness.
BenSalem [22]	Tunisia	Randomized placebo-controlled crossover trial	Well-trained female athletes (combat sports)	*n* = 15	Naturally menstruating athletes in different menstrual phases	Acute ingestion of 1.5 g acetaminophen	Placebo	Repeated high-intensity shuttle-run performance (5mSRT)	Pain perception, perceived exertion, recovery, delayed-onset muscle soreness, mood state, attention	Acetaminophen ingestion improved repeated sprint performance, mood state, attention, perceived recovery, and pain-related outcomes while reducing perceived exertion and muscle soreness.

**Table 2:** Risk of bias assessment of included studies using RoB 2 *.

Author	Selection	Assignment	Blinding	Full Data	Outcomes	Global
Bisgaard et al. [18]	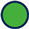	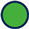	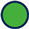	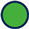	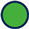	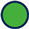
Messaoudène et al. [19]	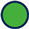	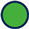	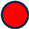	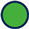	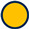	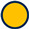
SantaBarbara et al. [20]	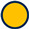	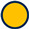	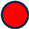	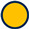	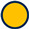	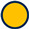
Safari et al. [21]	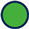	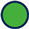	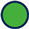	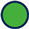	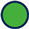	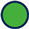
BenSalem et al. [22]	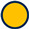	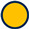	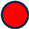	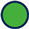	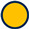	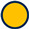

* Cochrane risk of bias tool for randomized trials. Risk Level: Green: Low, Yellow-Moderate, Red: High.

## Data Availability

No new data were created or analyzed in this study. Data sharing is not applicable to this article.

## Supplementary Materials

The following supporting information can be downloaded at: https://www.mdpi.com/article/10.3390/sports14060236/s1, Table S1: Prisma checklist.

## Abbreviations

The following abbreviations are used in this manuscript:

PRISMA	Preferred Reporting Items for Systematic Reviews and Meta-Analyses
DOMS	Delayed muscle soreness
GRADE	Grading of recommendations, assessment, development, and evaluations
